# Molecular diagnosis of somatic overgrowth conditions: A single‐center experience

**DOI:** 10.1002/mgg3.536

**Published:** 2019-02-13

**Authors:** Emilie Lalonde, Jessica Ebrahimzadeh, Keith Rafferty, Jennifer Richards‐Yutz, Richard Grant, Erik Toorens, Jennifer Marie Rosado, Erica Schindewolf, Tapan Ganguly, Jennifer M. Kalish, Matthew A. Deardorff, Arupa Ganguly

**Affiliations:** ^1^ Genetic Diagnostic Laboratory, Department of Genetics University of Pennsylvania Philadelphia Pennsylvania; ^2^ Penn Genomic Analysis Core, Perelman School of Medicine University of Pennsylvania Philadelphia Pennsylvania; ^3^ Center for Fetal Diagnosis and Treatment Children’s Hospital of Philadelphia Philadelphia Pennsylvania; ^4^ Division of Human Genetics Children’s Hospital of Philadelphia Philadelphia Pennsylvania; ^5^ Department of Pediatrics, Perelman School of Medicine University of Pennsylvania Philadelphia Pennsylvania

**Keywords:** mosaicism, *PIK3CA*‐related overgrowth spectrum, somatic overgrowth

## Abstract

**Background:**

Somatic overgrowth conditions, including Proteus syndrome, Sturge–Weber syndrome, and *PIK3CA*‐related overgrowth spectrum, are caused by post‐zygotic pathogenic variants, result in segmental mosaicism, and give rise to neural, cutaneous and/or lipomatous overgrowth. These variants occur in growth‐promoting pathways leading to cellular proliferation and expansion of tissues that arise from the affected cellular lineage.

**Methods:**

We report on 80 serial patients evaluated for somatic overgrowth conditions in a diagnostic laboratory setting, including three prenatal patients. In total, 166 tissues from these 80 patients were subjected to targeted sequencing of an 8‐gene panel capturing 10.2 kb of sequence containing known pathogenic variants associated with somatic overgrowth conditions. Deep next‐generation sequencing was performed with the IonTorrent PGM platform at an average depth typically >5,000×.

**Results:**

Likely pathogenic or pathogenic variants were identified in 36 individuals and variants of unknown significance in four. The overall molecular diagnostic yield was 45% but was highly influenced by both submitted tissue type and phenotype. In the prenatal setting, two patients had pathogenic variants identified in cultured amniocytes but in a third patient, the pathogenic variant was only present in post‐natal tissues. Finally, expanding the test to include full gene sequencing of *PIK3CA* in contrast to targeted sequencing identified likely pathogenic variants in 3 of 7 patients that tested negative on the original panel.

**Conclusion:**

Next‐generation sequencing has enabled sensitive detection of somatic pathogenic variants associated with overgrowth conditions. However, as the pathogenic variant allele frequency varies by tissue type within an individual, submission of affected tissue(s) greatly increases the chances of a molecular diagnosis.

## 1 INTRODUCTION

A heterogeneous group of overgrowth conditions are caused by post‐zygotic pathogenic variants, resulting in segmental mosaicism and give rise to neural, cutaneous and/or lipomatous overgrowth (Akgumus, Chang, & Li, 2017; Chang et al., 2017; Keppler‐Noreuil, Parker, Parker, Darling, & Martinez‐Agosto, 2016). Typically, these variants arise in growth‐promoting pathways, most commonly the PI3K‐AKT‐MTOR pathway, leading to cellular proliferation and enlargement of tissues arising from the affected cellular lineage. These conditions are not heritable (though germline variants exist in rare cases), and vary extensively in phenotype distribution and severity depending on which stage in embryogenesis the de novo variant arises, the particular cell of origin, and the type of variant (Mirzaa et al., 2016).

Somatic mosaicism for embryonic lethal pathogenic variants was long‐suspected to be responsible for diseases with segmental phenotypes (Happle, 1987), and non‐tumorigenic somatic variants were first identified in *GNAS* (OMIM 139320) in individuals with McCune–Albright syndrome in 1991 (Weinstein et al., 1991). Since then, post‐zygotic pathogenic variants in several genes have been linked to disease: *AKT1* (OMIM 164730) and Proteus syndrome, *PIK3CA* (OMIM 171834) and CLOVES syndrome (Congenital Lipomatous Overgrowth, Vascular malformations, and Epidermal nevi Scoliosis/skeletal/spinal anomalies), *GNAQ* (OMIM 600998) and Sturge–Weber syndrome, as well as many related phenotypes (Biesecker & Spinner, 2013; Lindhurst et al., 2011; Riviere et al., 2012). Pathogenic post‐zygotic *PIK3CA* variants are associated with additional mosaic syndromes and isolated findings, now collectively termed *PIK3CA*‐related overgrowth spectrum (PROS) (Keppler‐Noreuil et al., 2015). Discovery of these somatic changes has been facilitated by deep next‐generation sequencing (NGS), which can detect extremely low‐level mosaicism that could not have been identified by traditional sequencing methods or PCR‐based assays (Chang et al., 2017; Hucthagowder et al., 2017). Interestingly, these same variants also commonly occur in tumorigenesis, giving a proliferative advantage to the malignant clone. Thus, the same pathways driving malignancy also result in non‐cancerous cellular proliferation.

Here, we report on 80 consecutive patients evaluated for somatic overgrowth conditions in a diagnostic laboratory setting. We performed ultra‐deep sequencing of 25 exons with known pathogenic variants across eight genes in a variety of tissue types and sought to identify factors that influence diagnostic yield. For seven patients that tested negative but were highly suspicious of PROS, an expanded panel including the entire coding sequence of *PIK3CA* was evaluated.

## 2 MATERIAL AND METHODS

### 2.1 Ethical compliance

The study was deemed exempt from IRB approval based on use of samples that were collected from diagnostic samples which were submitted for genetic testing. This is according to the recommendations of the Office of Human Research Protections decision charts (https://www.hhs.gov/ohrp/regulations-and-policy/decision-charts/index.html#c5). All identifiers have been removed such that subjects cannot be identified.

### 2.2 Clinical case series

Patients were referred to the Genetics Diagnostic Laboratory by ordering physicians due to suspicion of a somatic overgrowth condition between November 2014 and July 2017. All cases received were included in the study, resulting in a case series of 80 individuals (Supporting information Table S1). Clinical indications for testing were typically obtained from test requisition forms, but were not always available or complete. Submitted samples were denoted as affected or unaffected for the majority of patients, and best judgment was used for the remaining patients based on the submitted specimen and clinical descriptions. For each submitted specimen, two distinct DNA isolations were processed independently and sequenced in separate batches for variant validation.

### 2.3 Somatic Overgrowth (OVG) and PIK3CA panel content

The OVG V1 panel utilized a targeted approach to interrogate 10.2 kb comprising 25 exons and flanking intronic sequences across 8 overgrowth‐associated genes: *PIK3CA *(NM_006218.2), *MTOR *(NM_004958.3), *PIK3R2 *(NM_005027.2), *AKT1 *(NM_005163.2), *AKT2 *(NM_001626.3), *AKT3 *(NM_005465.4), *GNAQ *(NM_002072.4), and *CDKN1C *(NM_000076.2). Collectively, these exons of interest harbored 27 hotspot loci at which pathogenic variants associated with overgrowth spectrum had been reported by the time of panel design in 2013 (Supporting information Table S2; Figure S1). Based on curated variants in Human Gene Mutation Database (v2017.4), 85% of reported variants have been found within these exons. In total, 24 reported variants were not captured by this panel, including 7 in *PIK3CA* (all of which are captured in the PIK3CA panel, below) and 9 in *MTOR*. However, 17 of these 24 variants were reported after panel design in 2013, and the remainder are germline changes found in inconsistent phenotypes (e.g., diabetes, isolated epilepsy, Cowden‐like syndrome).

The PIK3CA test covered 8.0 kb, including all 20 coding exons of *PIK3CA* and all flanking (20 bps) intronic regions. The untranslated exon 1 of *PIK3CA* was not covered by the panel. Of note, the *PIK3CA*‐specific NGS test covered 11 exons not covered by the OVG panel, corresponding to an increase of 2.2 kb of additional sequence coverage for *PIK3CA*.

### 2.4 DNA isolation

DNA was extracted from unaffected and affected samples using tissue‐appropriate protocols. Genomic DNA from peripheral blood lymphocytes was isolated using one of the following: the whole blood isolation protocol and reagents as described in the Gentra Puregene Handbook (Qiagen), the QIAamp DNA Blood Mini Isolation kit (Qiagen), or the Chemagen DNA Blood Kit and MSM I instrument (PerkinElmer). Genomic DNA from tissue biopsies was isolated following the “DNA Purification from Tissues materials” protocol for the QIAamp DNA Mini and Blood Mini Kit (Qiagen). Saliva samples were collected using the Oragene collection kit (DNA Genotek), and DNA was isolated using the prepIT‐L2P extraction kit (DNA Genotek). DNA from buccal samples was isolated using the Xtreme DNA Kit (Isohelix). DNA from cultured amniocytes was isolated using the Gentra Puregene Handbook (Qiagen). DNA from direct amniocytes was isolated using the QIAamp DNA Micro Kit and protocol (Qiagen). Extracted DNA was quantified using the Infinite M200 NanoQuant (Tecan).

### 2.5 Library preparation, templating, and sequencing

For the OVG panel, 21 of 25 target regions were amplified via a single multiplex reaction. Four remaining regions were amplified in individual reactions requiring different reaction parameters than that of the multiplex reaction. The minimum DNA input per amplicon is 14.25 ng. The multiplex and individual amplicons were pooled together at a ratio shown to produce optimal coverage across all target regions, and then quantified using a 2100 Bioanalyzer (Agilent) prior to library preparation. Sequencing libraries were synthesized using the Ion Shear™ Plus Reagents Kit (Life Technologies) and Ion Xpress™ Plus Fragment Library Kit (Life Technologies) following the manufacturer's recommended protocol. Briefly, 50 ng of pooled amplicons was used as template for enzymatic shearing. Sheared amplicons are ligated to Ion Xpress™ Barcode Adapters (Life Technologies) and P1 adapters, and then, the library was amplified a final time.

The full gene PIK3CA panel was prepared using the Ion AmpliSeq Library Kit 2.0‐384LV (Life Technologies) following the recommended protocol for a 3‐pool, half‐reaction library preparation as described in the Ion AmpliSeq Library Kit 2.0 User Guide, Revision E. Target regions were amplified via three multiplex reactions (3 primer pools), and subsequently combined into a single pool. Amplicons were digested, ligated with Ion Xpress™ Barcode Adapters (Life Technologies) and P1 adapters, and amplified a final time.

Final OVG and PIK3CA libraries were quantified using a 2100 Bioanalyzer (Agilent) and High Sensitivity Kits (Agilent), then diluted to 100 pmol/L for templating. Templating was performed using the ION PGM HI‐Q View OT2 Kit (Life Technologies) and DB MYONE Streptavidin C1 Beads (Life Technologies) following the manufacturer's recommended protocols. Libraries were sequenced on the ION PGM 318 v2 Chip (Life Technologies) with the ION PGM HI‐Q View Sequencing Kit (Life Technologies) using recommended procedures on the Ion Torrent PGM platform. Variants were confirmed by sequencing an independent replicate on the same NGS platform.

### 2.6 NGS data pipeline

Next‐generation sequencing data were processed by Torrent Suite software (versions v4.2.1.4 – 5.04). Raw signal data were processed, followed by base calling resulting in unaligned BAM files. Unaligned reads were aligned to the hg19 reference genome using the Torrent Mapping Alignment Program prior to variant calling with the Torrent Variant Caller (versions 4.2.1.4 – 5.04). The default Somatic‐Low Stringency parameters of the PGM platform were used for variant calling (Supporting information Table S3). Coverage analysis to assess depth of coverage at both a target and target base level was performed (Coverage Analysis plugin—versions 4.2 – 5.04). Variants present in two technical replicates were manually curated and classified according to the 2015 American College of Medical Genetics guidelines (Richards et al., 2015). Patients with a variant of unknown significance (VUS) (*n* = 4) were excluded from analysis.

### 2.7 Coverage performance

In the OVG Panel, coverage depth at the targeted hotspots achieved ≥2,000×. The mean base coverage depth across all targeted bases typically exceeded 5,000×, and 100% of target regions had ≥500 reads. Moreover, with exception of exon 1 of *CDKN1C*, 100% of bases in the 25 targeted exons were covered at ≥500×. Exon 1 of *CDKN1C* was Sanger sequenced to compensate for low coverage at the 3’ end of exon 1.

The mean base coverage depth of the PIK3CA full gene panel typically exceeded 5000x, and 100% of amplicons had ≥500 reads. All target bases in the panel were covered at ≥500×. The panel achieved 100% uniformity, defined as the proportion of target bases covered by 20% of the mean coverage. For both panels, reads were down‐sampled to 2,000× (Supporting information Table S3).

### 2.8 Statistical analysis

Data summarization and visualization was performed using the R statistical language (v3.3.1) and the ggplot2 library (v2.2.1). PIK3CA variants were visualized with the MutationMapper software from the cBio data portal (Cerami et al., 2012; Gao et al., 2013).

## 3 RESULTS

### 3.1 Diagnostic yield and clinical indication

**Figure 1:**
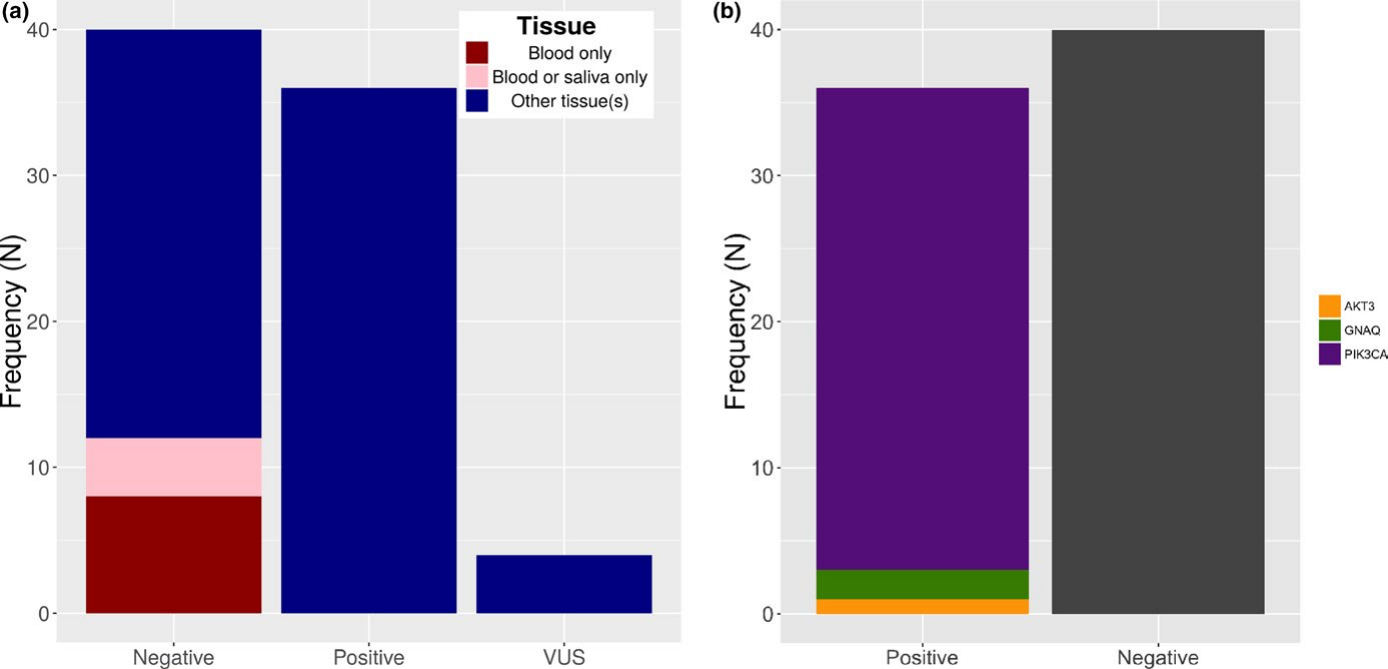
Pathogenic or likely pathogenic variant detection rate. (a) The diagnostic rate was 45% in this case series and was influenced by sample type. (b) The majority of likely pathogenic or pathogenic variants were identified in *PIK3CA* (GenBank Accession NM_006218.2)

We applied the OVG panel to 166 specimens from 80 individuals suspected of having an overgrowth condition (phenotypes listed in Supporting information Table S1). These patients were referred to the Genetics Diagnostic Laboratory by ordering clinicians. Overall, we identified pathogenic or likely pathogenic variants in 36 individuals, and VUS in four individuals (Figure 1), resulting in an overall diagnostic rate of 45% (36/80). The four individuals with VUS (one in *AKT1* and *PIK3CA* each, and two in *AKT2*) were removed from subsequent analysis to avoid bias in either direction. These VUS were identified in all submitted specimens for each individual, including blood, with a variant allele frequency (VAF) near 50%, and were present in population control databases at a greater frequency than expected for somatic overgrowth conditions. The remainder of the manuscript assesses 76 individuals with pathogenic, likely pathogenic or negative results. Of the pathogenic or likely pathogenic variants (P/LP variants) identified in the 36 positive patients, the vast majority (34/38) were in *PIK3CA*, two were in *GNAQ*, and one was in *AKT3*. In one instance, three distinct P/LP variants were identified in an individual (see below). The variants identified in these patients are listed in Supporting information Table S4.

**Figure 2:**
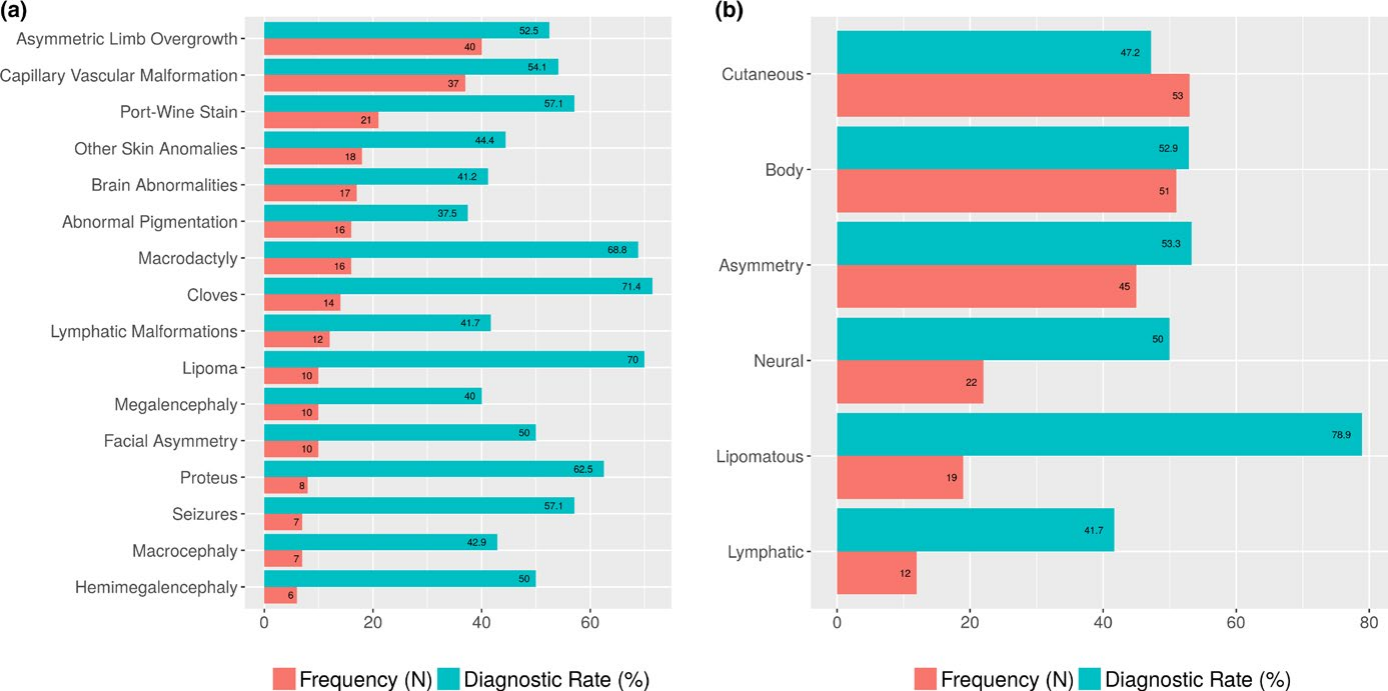
Frequency of the most common test indications (a) and symptom groups (b). For example, asymmetric limb overgrowth was the most common phenotype, and a suspicion of CLOVES syndrome or lipomatous findings were associated with the highest diagnostic yields

Chart review of the 76 informative patients revealed that the most common test indications were asymmetric limb overgrowth, capillary or vascular malformations, and macrodactyly (Figure 2a, Supporting information Table S5). A differential was included for 21/76 patients, including CLOVES, Proteus syndrome, megalencephaly‐capillary malformation syndrome (MCAP), PROS, Beckwith–Wiedemann Syndrome, and Sturge–Weber syndrome. PROS itself is an umbrella term that encapsulates a number of the clinical phenotypes submitted and others, including fibroadipose hyperplasia, hemihyperplasia multiple lipomatosis, CLOVES, MCAP, and dysplastic megalencephaly (Keppler‐Noreuil et al., 2015). Clinical suspicion of CLOVES or Proteus resulted in high diagnostic rates, of 71.4% and 62.5%, respectively, but interestingly, all of these patients were positive for *PIK3CA* P/LP variants (seven patients had CLOVES as the only differential, while 6 had additional differentials including PROS). Functional grouping of symptoms revealed that lipomatous symptoms (e.g., lipoma, suspicion of CLOVES) had the highest diagnostic rate, 79%, and were present in 19 of 76 patients (Figure 2b).

### 3.2 Tissue heterogeneity

**Figure 3:**
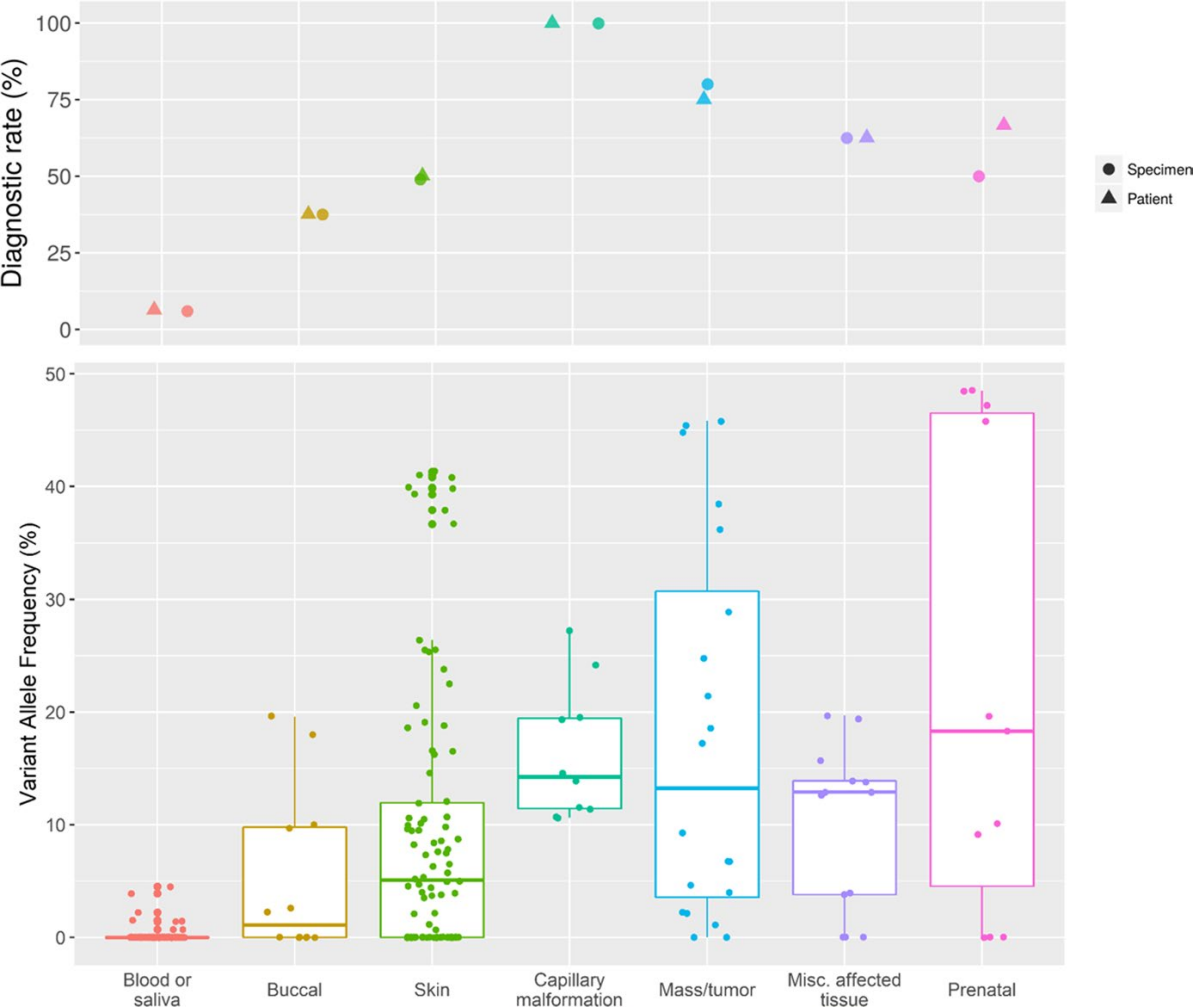
Diagnostic rate and variant allele frequency observed per specimen type. Diagnostic rate per patient and per specimen type are closely related (top panel). Variant allele frequency observed in 36 positive patients' (bottom panel), where each. Each value corresponds to one technical replicate per specimen

We request 1–2 affected and one unaffected specimen per patient, with an assumption that any putative P/LP variant will be absent, or present at very low variant allele frequency, in the unaffected sample (typically blood), allowing for somatic classification and increased confidence in pathogenicity. On average, each individual had two tissues submitted for analysis, where 45 patients had a blood sample and at least one other tissue, 14 patients had one or more non‐blood/saliva tissues (including three prenatal patients), and 17 patients had blood/saliva only. The most common sample type was blood (*n* = 62), followed by skin (*n* = 60, including five tissues specifically denoted as capillary malformations), and tumors or masses (*n* = 10). The variant allele frequency observed in the 36 positive patients ranged from 0.7% to 48.5%, consistent with mosaic variants (Figure 3). Excluding prenatal samples, the tissues with the highest VAF were tumors or other benign masses, capillary malformations, and a subset of skin samples, including some cultured fibroblasts and some affected skin specimens such as capillary malformations. Other miscellaneous affected tissues also had relatively high VAF, and included specimens such as muscle, brain biopsies and subcutaneous tissue. Conversely, variants present below 5% were detected in 13 patients, illustrating the importance of very deep sequencing.

Importantly, for all 12 patients where only blood (*n* = 8) or blood and saliva (*n* = 4) were submitted, our diagnostic rate was 0%. Moreover, blood was submitted for 28/36 positive patients, and the variant was detectable in DNA extracted from blood for only 4 patients, including 1 where the VAF was below the lab‐defined limit of detection but was observed by manual inspection of reads in IGV. The maximum VAF of a P/LP variant in blood was 4.5%. These results reinforce previous findings that testing blood alone is not a suitable strategy for diagnosis of somatic overgrowth, although some exceptions have been noted (Riviere et al., 2012, Keppler‐Noreuil et al., 2015, Luks et al. 2015, Mirzaa et al., 2016, Chang et al., 2017, Kuentz et al., 2017). The diagnostic yield increased to 56% (36/64) after removing patients where only blood and/or saliva were submitted.

Ideally, the submitted tissue would be taken from a clearly affected tissue; however, in many patients, these may be difficult or impossible to obtain. We thus sought to determine whether saliva, buccal, or skin biopsies are suitable alternatives to blood when clearly affected tissue is not available. P/LP variants were detectable in 0/4, 3/8, and 20/40 in individuals with submitted saliva, buccal, and skin samples, respectively. Of the 20 individuals with a variant identified in skin, the variants were identified in 19 “affected” skin samples (corresponding to 25/40 “affected” skin samples overall since some patients had multiple affected skin samples), compared to only 2/12 “unaffected” skin samples. These results illustrate that in the absence of a clearly affected tissue, skin biopsies and buccal swabs may be used as alternatives; however, the diagnostic yield is considerably lower compared to submitting a clearly affected tissue.

**Figure 4:**
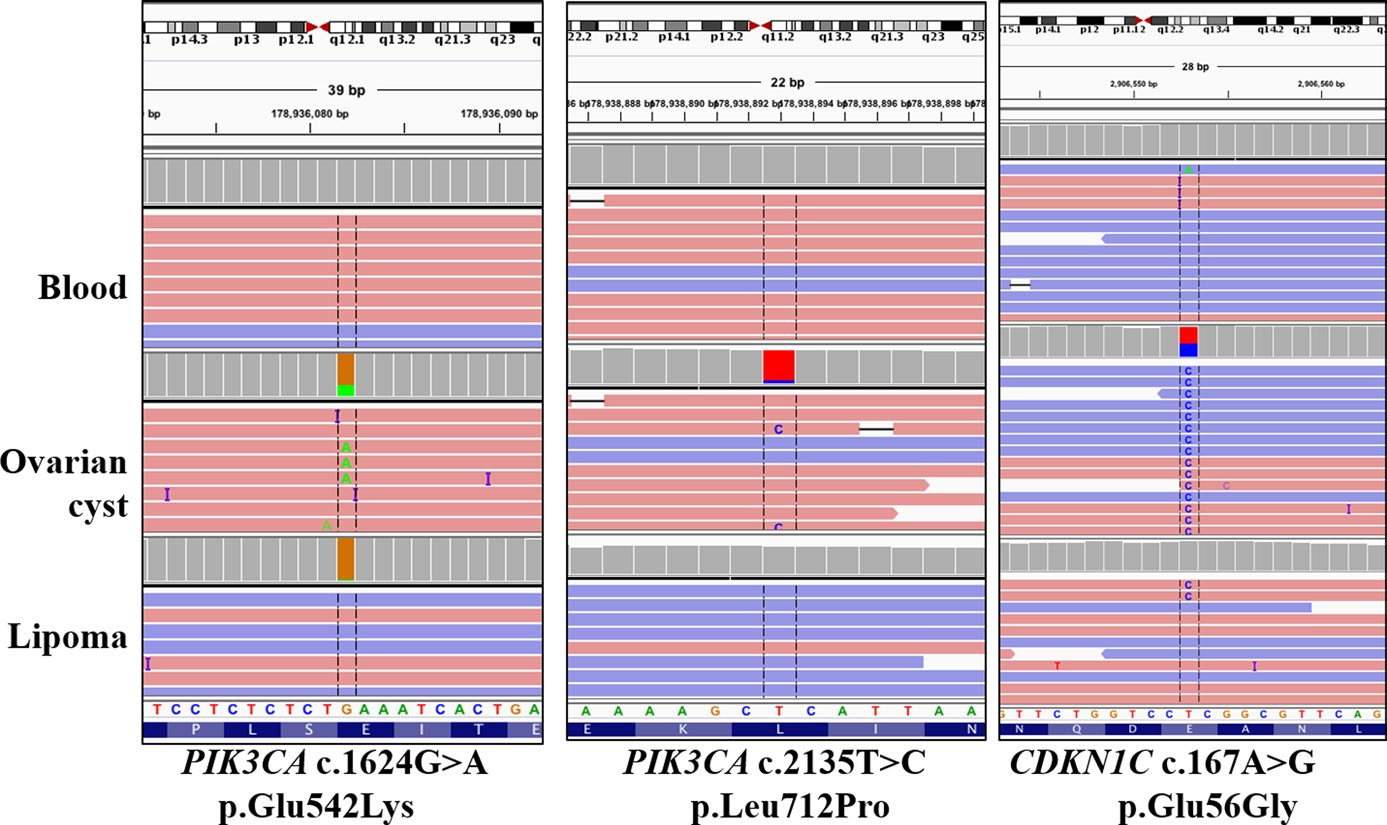
IGV screenshot for pathogenic variants observed in distinct tissues from one individual

Genetic heterogeneity was observed in a woman with multiple congenital lipomata, vascular malformations, and an ovarian cyst. A lipoma excised from the chest and an ovarian cyst were submitted for analysis (Table 1, Figure 4). Both the lipoma and the ovarian cyst had a *PIK3CA* pathogenic variant, c.1624G>A p.(Glu542Lys), present at less than 5% in the lipoma and at 29%–38% in the ovarian cyst. Remarkably, the ovarian cyst also had a second likely pathogenic variant in *PIK3CA*, c.2135 T>C (p.(Leu712Pro), VAF <10%), and a third likely pathogenic variant in *CDKN1C*, c.167A>G (p.(Glu56Gly), VAF 36%–45%). All three variants were absent in the patient's blood, indicating they are somatic in origin.

**Table 1:** Distinct likely pathogenic or pathogenic variants observed in multiple tissues from one individual

	Blood	Chest lipoma	Ovarian cyst	ClinVar	COSMIC	Classification
*PIK3CA* c.1624G>A p.(Glu542Lys)	0.16%–0.17% (13/8,307, 15/8,495)	2.5%–4.1% (195/7,952, 211/5,089)	26.7%–37.6% (3,876/14,524, 6,836/18,174)	Pathogenic ‐likely pathogenic (somatic, cancer and PROS)	>1,000 entries	Pathogenic
*PIK3CA* c.2135 T>C p.(Leu712Pro)	0.38%–0.40% (560/146,696, 465/115,368)	0.21%–0.29% (11/5,239, 32/11,184)	4.2%–9.6% (2,448/58,351, 2062/21,422)	Absent	1 entry (intestine)	Likely pathogenic
*CDKN1C* c.167A>G p.(Glu56Gly)	0.19%–0.23% (87/45,141, 101/44,213)	0%–2.2% (0/110, 2/93)	35.2%–45.0% (82/233, 85/189)	Absent	Absent	Likely pathogenic

Note

For each variant in each tissue, the variant allele frequency observed in two technical replicates is listed, followed by the ratio of reads with the variant in each replicate as observed in the Integrative Genome Viewer (IGV)

Based on *PIK3CA* GenBank Accession NM_006218.2

COSMIC: Catalogue of Somatic Mutations in Cancer; PROS: PIK3CA‐related overgrowth spectrum

### 3.3 Prenatal diagnosis

Of three prenatal cases submitted for evaluation, two were positive for *PIK3CA* pathogenic variants, and the third had a diagnostic finding on post‐natal tissue only (Table 2). In the first case, CLOVES was suspected based on lymphatic dysplasia, macrodactyly, and skin anomalies. Initial testing on DNA extracted from direct uncultured amniocytes did not reveal any P/LP variants. However, cultured amniocytes were sent shortly afterward, where a pathogenic *PIK3CA* variant was identified with a VAF 9.1%–19.6%. Retrospective analysis of the NGS reads from uncultured amniocytes at this site revealed the variant, but at levels significantly below the lab‐defined limit of detection where its initial detection could not be distinguished from background.

**Table 2:** Summary of prenatal findings. All variants were identified in *PIK3CA *(GenBank Accession NM_006218.2)

Case	Prenatal findings	Tissue	Genetic results	VAF (%)
1	Differential: CLOVES Macrodactyly, lymphedema and lymphatic malformations	Direct amniocytes	Below lab‐defined limit of detection	0.23%–0.31%
		Cultured amniocytes	c.3140A>G p.(His1047Arg)	9.1%–19.6%
2	Differential: MCAP Hemihypertrophy, cranial asymmetry, facial asymmetry, hemimegalencephaly, ventriculomegaly, macrodactyly, capillary/vascular malformations, agenesis of corpus callosum, polymicrogyria, frontal bossing, micrognathia, hypertelorism, skin thickening, subcutaneous edema of back, shortened tibia‐fibula, abnormal toes including some missing and some larger.	Cultured amniocytes	c.1624G>A p.(Glu542Lys)	45.8%–48.5%
3	Differential: CLOVES or Proteus Hemihypertrophy, megalencephaly, LGA (fetal size >97%), skin anomalies, concern for lymphedema and overgrowth, hypoechoic areas on ultrasound, abnormally dilated vessels in neck/chest, neck mass, cutaneous lesions on legs Post‐natal: Facial asymmetry, port‐wine stain, macrodactyly, hyperpigmentation, CLOVES syndrome	Cultured amniocytes	Below lab‐defined limit of detection	0.067%–0.19%
		Post‐natal chest skin biopsies (*n* = 2)	c.1633G>A p.(Glu545Lys)	7.8%–8.4%; 18.8%–20.6%
		Post‐natal blood	Below lab‐defined limit of detection	0.13%–0.34%

Note

CLOVES: Congenital Lipomatous Overgrowth, Vascular malformations, Epidermal nevi and Scoliosis/skeletal/spinal anomalies; LGA: Large for Gestational Age; MCAP: Megalencephaly‐Capillary Malformation; VAF: Variant Allele Frequency.

The second prenatal case had multiple findings from first trimester ultrasound and MRI, including overgrowth symptoms involving the body and head suggestive of MCAP. A *PIK3CA* c.1624G>A p.(Glu542Lys) pathogenic variant was identified with a VAF of 45.8%–48.5% in cultured amniocytes. It is not clear whether this high VAF is representative of a very early post‐zygotic variant or whether the variant was selected for during culture. Several cases of germline *PIK3CA* variants have been reported recently (Chang et al., 2017; Mirzaa et al., 2016). Ultimately, the pregnancy did not go to full‐term.

For the third prenatal case, no variant was identified in cultured amniotic fluid despite phenotypes highly consistent with a somatic overgrowth disorder. Post‐natal testing of two chest skin biopsies within 2 cm of one another revealed a pathogenic *PIK3CA* variant, c.1633G>A p.(Glu545Lys), at 7.8%–8.4% in one biopsy, and 18.8%–20.6% in the other, indicating genetic heterogeneity within the affected tissue. Indeed, the two skin biopsies contained regions of port‐wine stain and hypopigmentation. This variant was observed at extremely low levels in the blood of the neonate, well below the lab‐defined limit of detection (0.13%–0.34%, corresponding to 15/11,441 and 30/8,743 reads in the two replicates, respectively). Similarly, visual re‐examination of the prenatal sequencing data revealed this variant present at levels below the lab‐defined limit of detection (0.067%–0.19%, corresponding to 60/89,259 and 102/52,318 reads, respectively). It is important to note that while this variant can be detected at extremely low VAF, it could not be confidently identified a priori without the skin biopsy results. At this VAF, the false positive rate increases due to background noise.

### 3.4 Exploration of negative findings

Finally, we evaluated the likely cause of negative findings in the 40 patients where a P/LP variant was not identified. As mentioned previously, 12/40 of these patients only had blood available for testing, and thus, it is likely that, for a subset of these patients, P/LP variants were present in the targeted regions but not detectable in blood (Riviere et al., 2012, Keppler‐Noreuil et al., 2015, Luks et al., 2015, Mirzaa et al., 2016, Chang et al., 2017, Kuentz et al., 2017). To determine whether full *PIK3CA* sequencing would increase the molecular diagnostic rate, we evaluated an expanded panel which included full exonic coverage for *PIK3CA* in 7 patients where clearly affected tissue was submitted and the phenotype was strongly suggestive of PROS (Supporting information Table S6). Three likely pathogenic variants were identified in these patients, including two individuals with the same likely pathogenic variant, p.(Gly118Asp). Despite the small sample size, these results indicate that full *PIK3CA *gene sequencing is warranted for diagnosis of somatic overgrowth conditions.

Therefore, the number of patients with unexplained findings is reduced to 25/76. It is possible that these patients have other *PIK3CA* P/LP variants, not covered by the original panel, or have different genetic etiologies not captured by our current panels. For example, Patient 42 later tested positive for Beckwith–Wiedemann syndrome due to uniparental isodisomy for 11p15.5. The submitted phenotypes with the lowest diagnostic rates (not necessarily in isolated settings) include large for gestational age (0%, *n* = 4), organomegaly (0%, *n* = 2), generalized overgrowth (0%, *n* = 1), hyperinsulinism (0%, *n* = 1), neonatal hypoglycemia (20%, *n* = 5), and Klippel–Trenaunay syndrome (25%, *n* = 4).

### 3.5 *PIK3CA* analysis

**Figure 5:**
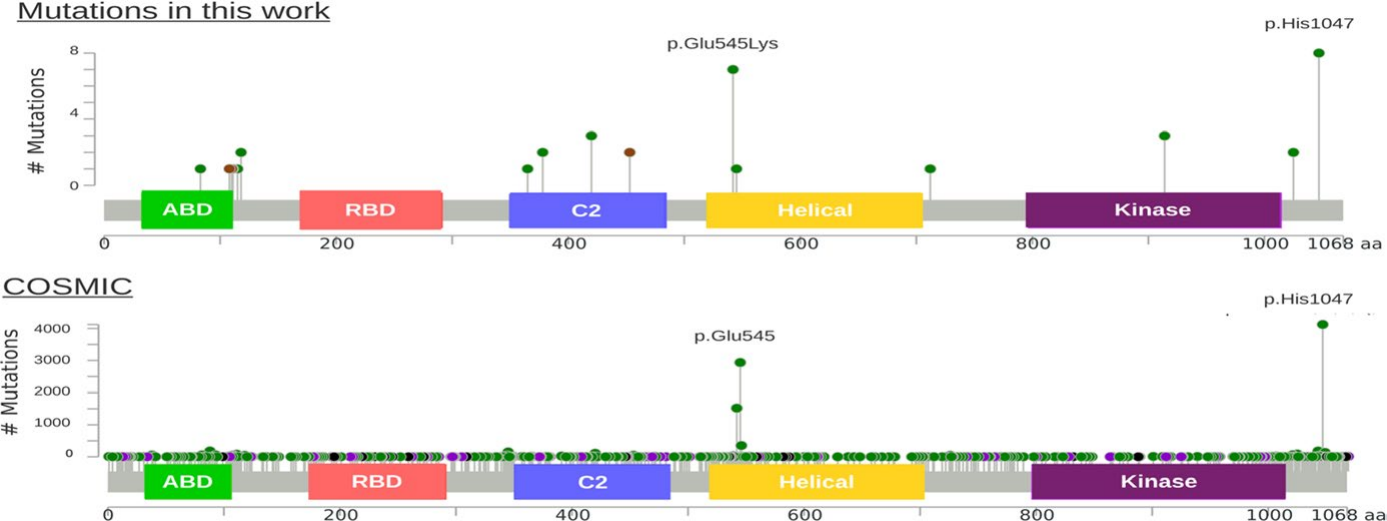
PIK3CA variants identified in the current study (top) and in the COSMIC database (bottom). Missense changes are shown in green, truncating changes in black, in‐frame indels in brown, and “other changes” in purple. The domains shown are the adaptor‐binding domain (green), the Ras‐binding domain (red), the membrane‐binding domain (blue), the helical domain (yellow), and the kinase catalytic domain (purple). COSMIC mutations accessed August 22, 2017. Nomenclature in reference to GenBank Accession NM_006218.2

Overall, 36 of the 39 P/LP variants identified in this study (including full *PIK3CA* sequencing results) occurred in *PIK3CA*, resulting in PROS. Our initial testing strategy captures exons including known P/LP variants at the time of panel design, but was expanded to full gene coverage in a subset of 7 patients. P/LP variants identified included both known hotspot and rare variants (Figure 5). For example, one likely pathogenic variant, p.(Lys111Glu), was present at 17.2%–18.6% in a granuloma from a patient with hemihypertrophy, macrodactyly, hyperpigmentation, and with a suspicion of CLOVES syndrome. This residue occurs just after the PIK3CA adaptor‐binding domain. It has never been reported in association with overgrowth syndromes, but appears 35 times in COSMIC, and other variants at this residue have also been observed, including different missense changes and in‐frame deletions. This likely pathogenic variant is not present in population databases.

Somatic variants along the entire length of *PIK3CA* have been identified in various cancers, including many of the same hotspot P/LP variants observed in PROS. There have been conflicting reports whether hotspot *PIK3CA* variants with strong oncogenic activity give rise to the MCAP phenotype and brain overgrowth (Kuentz et al., 2017; Mirzaa et al., 2016). In our study which is enriched for exons containing known pathogenic variants, a hotspot variant was identified in both patients submitted with MCAP as a potential diagnosis (p.(Glu542Lys) and p.(His1047His)). Additionally, of the fifteen individuals with a hotspot P/LP variant, four had symptoms highly suggestive of MCAP, including one prenatal case. These results reinforce the notion that multiple types of variants can cause both MCAP and non‐MCAP phenotypes but that there is an enrichment of non‐oncogenic hotspot variants giving rise to MCAP.

## 4 DISCUSSION

Molecular diagnosis for somatic overgrowth conditions has been historically challenging due to genetic heterogeneity, tissue specificity and low‐level mosaic P/LP variants (Keppler‐Noreuil et al., 2015). With the advent of NGS, efficient molecular diagnosis of somatic overgrowth conditions has become feasible and genetic testing has become available from clinical laboratories (Chang et al., 2017; Mirzaa et al., 2016). However, NGS does not completely address the challenge of tissue heterogeneity. Because mosaic variants arise post‐zygotically, only affected tissues will contain these variants at a significant VAF detectable by NGS. Testing blood samples severely limits the diagnostic efficiency (Riviere et al., 2012, Keppler‐Noreuil et al., 2015, Luks et al. 2015, Mirzaa et al., 2016, Chang et al., 2017, Kuentz et al., 2017); in our study, P/LP variants were identified by our bioinformatics pipeline in only 3 of 62 patients (in 1 additional patient a variant was observed in the raw sequence data below the lab‐defined limit of detection after detecting the variant in an affected tissue). All four of these individuals had capillary vascular malformations and body overgrowth, and three had neural symptoms consistent with MCAP or megalencephaly‐polymicrogyria‐polydactyly‐hydrocephalus (MPPH). This is in agreement with previous studies where individuals with identifiable pathogenic variants in blood have phenotypes consistent with MCAP and MPPH, which are presumed to affect earlier cell lineages (Chang et al., 2017; Kuentz et al., 2017). However, for the vast majority of patients, including those with severe phenotypes, testing blood will not identify a molecular cause, and as such, ordering physicians should avoid submitting only blood or saliva for molecular diagnosis of somatic overgrowth conditions when possible (Keppler‐Noreuil et al., 2015).

There is contradictory evidence regarding whether culturing tissue may increase the diagnostic yield. Kuentz and colleagues suggested that fresh skin tissue should be preferred over cultured fibroblasts, whereas Chang and colleagues found that culturing fibroblasts increases the VAF and thus the ability to detect the disease‐associated variant (Chang et al., 2017; Kuentz et al., 2017). In our experience, only eight cultured fibroblast samples were submitted, of which four were found to have a P/LP variant, ranging from 3%–49% VAF. We had no cases with matched direct and cultured fibroblasts to evaluate the effect of culturing, except in the prenatal setting (see below). While culturing fibroblasts can select for a growth‐promoting variant, it may introduce artifacts that are selected for and result in false positives. Genetic drift can also result in the loss of a variant, leading to false negatives, as observed for 3 patients by Kuentz and colleagues and in one of our prenatal patients (Kuentz et al., 2017). More studies involving matched direct and cultured fibroblasts are needed to resolve this issue.

In this study of 80 individuals suspected to have a somatic overgrowth condition, 39 molecular diagnoses were made (36 with the original panel, and 3 with the expanded panel) and four variants of unknown significance were identified. Excluding the 12 patients where only blood was submitted increases the diagnostic yield to 60.9% (39/64). This rate is comparable to prior studies where the molecular diagnostic yield varied from 33%–67% (Chang et al., 2017; Kuentz et al., 2017; Mirzaa et al., 2016). This wide range is likely due to different diagnostic strategies (full gene vs. targeted, *PIK3CA *only vs. panel), sequencing coverage, and differences in patient ascertainment and specimen submission. For instance, Kuentz and colleagues had strict clinical and specimen eligibility criteria, and found that diagnostic rates were higher in individuals with syndromic findings rather than isolated (Kuentz et al., 2017). Our diagnostic rate is likely to increase as we continue to sequence more patients with the expanded panel, which includes full *PIK3CA* sequencing. In our protocol, we request one unaffected tissue and up to two affected tissues. While sequencing unaffected tissues typically does not reveal a P/LP variant, it provides evidence of somatic variants, and increases the confidence in pathogenicity for any detected variant, which may be present at VAF as low as 2%. This strategy results in a relatively high diagnostic yield, and very low rate of VUS, compared to most diagnostic tests.

In one individual, three distinct P/LP variants were observed, including two co‐occurring *PIK3CA* variants in an ovarian cyst. The first pathogenic variant is the well‐known hotspot variant p.(Glu542Lys) which was also present in a chest lipoma biopsy, albeit at a lower frequency. The second *PIK3CA *likely pathogenic variant, p.(Leu712Pro), was found only in the ovarian cyst, and has not been reported in ClinVar or gnomAD but had been reported once in the COSMIC somatic variant database (http://cancer.sanger.ac.uk/cosmic). This variant was identified in a lung metastasis from an individual with pT4b colorectal cancer (Kovaleva et al., 2016). The functional consequence of this change is unclear, as it is not located in a protein domain, though at least one other nearby pathogenic variant, p.(Glu726Lys), has been documented in many patients with PROS (Chang et al., 2017; Kuentz et al., 2017; Mirzaa et al., 2016; Riviere et al., 2012). Additionally, a somatic p.(Glu56Gly) likely pathogenic was identified in *CDKN1C* that was similarly only present in the ovarian cyst. While we cannot determine the cause of three seemingly independent variants in one patient, it is possible that environmental or intrinsic pressure led to the acquisition of three distinct variants associated with segmental overgrowth. Alternatively, these variants could have evolved independently and or could even be indicative of a pre‐malignant state.

In the prenatal setting, a pathogenic variant was identified in all three prenatal cases and illustrated the utility of different tissue types for diagnosis. In one case, the variant was found in cultured but not direct amniocytes; in a second case, in cultured amniocytes only; and in the last case, the variant was only detectable in post‐natal skin biopsies. These variants were observed in all other tested tissues, but were below the lab‐defined limit of detection. One explanation for the tissue discrepancies between direct and cultured amniocytes is that this PIK3CA variant, p.(His1047Arg), provides a growth advantage in vitro, and is selected for by the culturing conditions. However, in the third case, the pathogenic variant, p.(Glu545Lys) which was found in post‐natal tissue, is also strongly oncogenic and was initially not identified in the cultured amniocytes; it is not clear whether it would have been identified in direct amniocytes. Chang and colleagues observed pathogenic variants in direct and cultured amniocytes of one fetus, and in the cultured but not direct amniocytes of a second fetus (Chang et al., 2017). Similarly, Emrick and colleagues reported a PIK3CA variant in cultured but not direct amniocytes of one fetus with a clinical diagnosis of CLOVES (Emrick et al., 2014). Until further studies are performed, it is recommended to test both direct (to avoid genetic drift) and cultured (to potentially enrich for affected cells) cells for prenatal diagnosis of suspected somatic overgrowth conditions, when possible.

In our case series, the majority of P/LP variants were identified in *PIK3CA*. Thus, if PROS is suspected, an efficient molecular diagnostic strategy would be to start with *PIK3CA* analysis and reflex to the larger somatic overgrowth panel if needed. However, clinical features may be ambiguous and if a rapid turnaround time is preferred, using the full panel as a first‐pass is preferable. For instance, pathogenic variants in *PIK3CA*, *PIK3R2*, *mTOR*, *AKT1*, *AKT3, *and *PTEN *can result in overlapping phenotypes. In general, many of these can be distinguished clinically but if only non‐specific features are present, the causative‐gene may not be obvious. The greatest clinical diagnostic challenge lies in patients with isolated asymmetry of a limb or hand/foot which could be due to any one of the aforementioned genes. In these patients, as demonstrated by the data presented here, there is clinical utility in testing skin from the affected limb.

Molecular diagnosis of a somatic overgrowth condition can help guide patient management, aid in family planning, and may offer therapeutic opportunities. For individuals with a P/LP variant in the PIK3CA‐AKT‐mTOR pathway, mTOR inhibitors and small molecular inhibitors targeting AKT or PIK3CA, initially developed for oncological purposes, are now showing promise in off‐label trials and pre‐clinical models for management of somatic overgrowth conditions (e.g., sirolimus: NCT02428296; ARQ 092: NCT03094832) (Akgumus et al., 2017; Chang et al., 2017; Keppler‐Noreuil et al., 2016).

Limitations of this study include incomplete phenotypic information for some patients, and being dependent on information submitted by providers. Additionally, our genotype–phenotype analysis is restricted to genetic regions tested (i.e., our OVG panel covers 9 of 21 exons for *PIK3CA*, but covers 61% of the protein domains, excluding the RNA binding domain which was not included in the design). For instance, MCAP features are enriched in individuals with non‐hotspot PIK3CA variants (Kuentz et al., 2017; Mirzaa et al., 2016). Nonetheless, 5/8 patients in our study which were highly suspicious of MCAP based on submitted phenotypes were found to have *PIK3CA *variants using the OVG panel, and a 6th patient had a likely pathogenic variant identified with the PIK3CA panel. Future studies will be performed with an expanded panel, including full *PIK3CA* gene sequencing, and additional genes associated with somatic overgrowth conditions.

In summary, recent technological advances have resulted in tremendous progress for molecular diagnosis of somatic overgrowth conditions. NGS enables the identification of variants present at very low levels (down to ~1% depending on methodology), which is critical given demonstrated tissue specificity of these post‐zygotic changes. Nonetheless, care must be taken to ensure that the most appropriate specimen is sent for analysis. Simultaneously, therapeutic progress for individuals with P/LP variants in the PIK3CA‐AKT‐mTOR pathway provides novel management strategies once a molecular diagnosis is made. Future studies will continue to define the best practices for the diagnosis and management of somatic overgrowth conditions.

## CONFLICTS OF INTEREST

None declared.
