# Supplementary material for: Molecular diagnosis of somatic overgrowth conditions: A single‐center experience
Source: Mol Genet Genomic Med. 2019 Feb 13;7(3):e536. doi: 10.1002/mgg3.536 (PMC6418364; doi:10.1002/mgg3.536)

# Supplemental Material

**Molecular Diagnosis of Somatic Overgrowth Conditions: A Single Center Experience**

Emilie Lalonde^1^, Jessica Ebrahimzadeh^1^, Keith Rafferty^1^, Jennifer Richards-Yutz^1^, Richard Grant^1^, Erik Toorens^2^, Jennifer Marie Rosado^2^, Erica Schindewolf^3^, Tapan Ganguly^2^, Jennifer M. Kalish^4,5^, Matthew A. Deardorff^4,5^, Arupa Ganguly^1^

1- Genetic Diagnostic Laboratory, Department of Genetics, University of Pennsylvania

2- Penn Genomic Analysis Core, Perelman School of Medicine, University of Pennsylvania

3- Center for Fetal Diagnosis and Treatment, Children’s Hospital of Philadelphia

4- Division of Human Genetics, Children’s Hospital of Philadelphia

5- Department of Pediatrics, Perelman School of Medicine, University of Pennsylvania

### Supplementary Tables

**Supplementary Table 1.** Patient demographics and clinical information. See additional file.

**Supplementary Table 2**. Target regions for the overgrowth v1 panel. (A) Summary (B) Full details

A)

| **Gene** | **Locus** | **RefSeq Transcript** | **Exons Covered:** | **No. of Targeted Hotspot Mutations** |
| --- | --- | --- | --- | --- |
| *PIK3CA* | 3q26.32 | NM_006218.2 | Exons 1, 5, 7-9, 13, 18-20 | 19 |
| *GNAQ* | 9q21.2 | NM_002072.4 | Exons 1-7 | 1 |
| *AKT1* | 14q32.33 | NM_005163.2 | Exon 2 | 1 |
| *AKT2* | 19q13.2 | NM_001626.3 | Exon 2 | 1 |
| *AKT3* | 1q43-q44 | NM_005465.4 | Exons 2, 7, 13 | 3 |
| *PIK3R2* | 19p13.11 | NM_005027.2 | Exon 9 | 1 |
| *MTOR* | 1p36.22 | NM_004958.3 | Exon 29 | 1 |
| *CDKN1C* | 11p15.4 | NM_000076.2 | Exons 1-2 | 0 |

B)

| Gene | Region of Interest | Locus |
| --- | --- | --- |
| *MTOR* | Exon 29 | chr1: 11217209 - 11217348 |
| *AKT3* | Exon 13 | chr1: 243668551 - 243668636 |
| *AKT3* | Exon 7 | chr1: 243776973 - 243777041 |
| *AKT3* | Exon 2 | chr1: 243858893 - 243859018 |
| *PIK3CA* | Exon 1 | chr3: 178916614 - 178916965 |
| *PIK3CA* | Exon 5 | chr3: 178922291 - 178922376 |
| *PIK3CA* | Exon 7 | chr3: 178927974 - 178928126 |
| *PIK3CA* | Exon 8 | chr3: 178928219 - 178928353 |
| *PIK3CA* | Exon 9 | chr3: 178935998 - 178936122 |
| *PIK3CA* | Exon 13 | chr3: 178938774 - 178938945 |
| *PIK3CA* | Exon 18 | chr3: 178947792 - 178947909 |
| *PIK3CA* | Exon 19 | chr3: 178948013 - 178948164 |
| *PIK3CA* | Exon 20 | chr3: 178951882 - 178952152 |
| *GNAQ* | Exon 7 | chr9: 80336239 - 80336429 |
| *GNAQ* | Exon 6 | chr9: 80343430 - 80343583 |
| *GNAQ* | Exon 5 | chr9: 80409379 - 80409508 |
| *GNAQ* | Exon 4 | chr9: 80412436 - 80412564 |
| *GNAQ* | Exon 3 | chr9: 80430532 - 80430686 |
| *GNAQ* | Exon 2 | chr9: 80537077 - 80537261 |
| *GNAQ* | Exon 1 | chr9: 80646016 - 80646151 |
| *CDKN1C* | Exon 2 | chr11: 2905234 - 2905364 |
| *CDKN1C* | Exon 1 | chr11: 2905900 - 2906719 |
| *AKT1* | Exon 2 | chr14: 105246425 - 105246553 |
| *PIK3R2* | Exon 9 | chr19: 18273777 - 18273957 |
| *AKT2* | Exon 2 | chr19: 40762833 - 40762961 |

**Supplementary Table 3.** Variant filtration parameters used for variant discovery

| **Parameter** | **Value** |
| --- | --- |
| data-quality-stringency | 10 |
| downsample-to-coverage | 2000 |
| filter-deletion-predictions | 0.2 |
| filter-insertion-predictions | 0.2 |
| filter-unusual-predictions | 0.12 |
| gen-min-alt-allele-freq | 0.01 |
| gen-min-coverage | 6 |
| gen-min-indel-alt-allele-freq | 0.01 |
| germline-prior-strength | 0 |
| heavy-tailed | 3 |
| hotspot-min-allele-freq | 0.02 |
| hotspot-min-cov-each-strand | 4 |
| hotspot-min-coverage | 100 |
| hotspot-min-variant-score | 6 |
| hotspot-strand-bias | 0.95 |
| hotspot-strand-bias-pval | 0.01 |
| hp-max-length | 8 |
| indel-min-allele-freq | 0.02 |
| indel-min-cov-each-strand | 4 |
| indel-min-coverage | 100 |
| indel-min-variant-score | 6 |
| indel-strand-bias | 0.9 |
| indel-strand-bias-pval | 1 |
| k-zero | 3 |
| max-complex-gap | 1 |
| max-detail-level | 0 |
| max-flows-to-test | 10 |
| min-delta-for-flow | 0.1 |
| minimum-sigma-prior | 0.085 |
| min-mapping-qv | 4 |
| min-ratio-reads-non-sse-strand | 0.2 |
| mnp-min-allele-freq | 0.02 |
| mnp-min-cov-each-strand | 4 |
| mnp-min-coverage | 100 |
| mnp-min-variant-score | 6 |
| mnp-strand-bias | 0.95 |
| mnp-strand-bias-pval | 0.01 |
| num-threads | 12 |
| num-variants-per-thread | 250 |
| outlier-probability | 0.005 |
| position-bias | 0.75 |
| position-bias-pval | 0.05 |
| position-bias-ref-fraction | 0.05 |
| prediction-precision | 1 |
| prefix-exclude | 6 |
| read-max-mismatch-fraction | 1 |
| read-rejection-threshold | 0.5 |
| read-snp-limit | 10 |
| realignment-threshold | 0 |
| shift-likelihood-penalty | 0.3 |
| sigma-prior-weight | 1 |
| slope-sigma-prior | 0.0084 |
| snp-min-allele-freq | 0.02 |
| snp-min-cov-each-strand | 4 |
| snp-min-coverage | 100 |
| snp-min-variant-score | 6 |
| snp-strand-bias | 0.95 |
| snp-strand-bias-pval | 0.01 |
| soft-clip-bias-checker | 0.1 |
| sse-prob-threshold | 1 |
| sse-relative-safety-level | 0.025 |
| tune-sbias | 0.01 |
| use-best-n-alleles | 2 |
| allow-complex | false |
| allow-indels | true |
| allow-mnps | true |
| allow-snps | true |
| do-json-diagnostic | false |
| do-minimal-diagnostic | false |
| do-mnp-realignment | false |
| do-snp-realignment | false |
| heal-snps | true |
| indel-as-hpindel | false |
| left-align-indels | false |
| override-limits | false |
| process-input-positions-only | false |
| resolve-clipped-bases | false |
| suppress-nocall-genotypes | true |
| suppress-no-calls | true |
| suppress-recalibration | false |
| suppress-reference-genotypes | true |
| use-input-allele-only | false |
| use-position-bias | false |
| use-sse-basecaller | true |

**Supplementary Table 4.** Variants of clinical significance identified in the study. See additional file.

**Supplementary Table 5.** Frequency and diagnostic rate of the most common test indications.

| **Symptom** | **Frequency (N)** | **Diagnostic rate (%)** |
| --- | --- | --- |
| **Neural** | 22 | 50 |
| Megalencephaly | 10 | 40 |
| Hemimegalencephaly | 6 | 50 |
| Brain abnormalities | 17 | 41.2 |
| **Body overgrowth** | 51 | 52.9 |
| Asymmetric limb growth | 40 | 52.5 |
| Facial asymmetry | 10 | 50 |
| Macrodactyly | 16 | 68.8 |
| Large for gestational age | 4 | 0 |
| **Cutaneous** | 53 | 47.2 |
| Port-wine stain | 21 | 57.1 |
| Abnormal pigmentation | 16 | 37.5 |
| Epidermal nevus | 3 | 100 |
| Capillary vascular  malformation | 37 | 54.1 |
| Other skin anomalies | 18 | 44.4 |
| **Lipomatous** | 19 | 78.9 |
| Lipoma | 10 | 70 |
| CLOVES | 14 | 71.4 |
| **Lymphatic malformations** | 12 | 41.7 |
| **Asymmetry** | 45 | 53.3 |
| Cranial asymmetry | 4 | 100 |
| Limb asymmetry | 40 | 52.5 |
| Facial asymmetry | 10 | 50 |
| **Others** |  |  |
| Seizures | 7 | 57.1 |
| Organomegaly | 2 | 0 |
| Macrocephaly | 7 | 42.9 |
| Macroglossia | 2 | 50 |
| CLOVES | 14 | 71.4 |

**Supplementary Table 6.** Cases analyzed with the PIK3CA panel after negative results with the OVG panel.

| **Case** | **Specimens** | **Phenotype** | ***PIK3CA* result** | **Mutation information** | **VAF (%)** |
| --- | --- | --- | --- | --- | --- |
| 15 | 1) Blood  2) Skin punch biopsy from left thigh | Port-wine stains, Klippel Trenaunay syndrome, asymmetric limb overgrowth (L>R), left-sided vascular anomalies  Suspect Proteus or CLOVES syndromes | Positive  Likely pathogenic | c.353G>A (p.Gly118Asp) | 11.9% in skin punch |
| 21 | 1) Blood  2) Left thigh skin punch biopsy | Asymmetric limb overgrowth, facial asymmetry, port-wine stain, macrodacyly, hyperpigmentation | Positive  Likely pathogenic | c.353G>A (p.Gly118Asp) | 4.8% in skin punch |
| 70 | 1) Blood  2) Affected skin punch biopsy | Asymmetric limb overgrowth (L >R, especially torso, left leg), absent corpus collosum, R Hemimegalencephaly, seizures.  Vascular malformation, lymphatic in appearance  Suspect Proteus or CLOVES syndromes | Positive  Likely pathogenic | c.321_323delCCG  (p.Arg108del) | 4.4% in skin punch |
| 3 | 1) Previously isolated DNA from toe biopsy | Suspected CLOVES | Negative | NA | NA |
| 20 | 1) Blood  2) Right foot biopsy  3) Left toe biopsy | Asymmetric limb overgrowth, facial asymmetry, macrodactyly, pulmonic stenosis, hyperpigmentation | Negative | NA | NA |
| 40 | 1) Blood  2) Previously isolated DNA from brain | Asymmetric limb overgrowth, hemimegalencephaly, linear hypopigmented lesion on nose | Negative | NA | NA |
| 65 | 1) Blood  2) Atrial biopsy | Bilateral post axial polysyndactyly, bilateral polymicrogyria and cortical dysplasia, hypotonia, macrosomia, tetralogy of fallot | Negative | NA | NA |

### Supplementary Figures

**Supplementary Figure 1.** UCSC screenshots demonstrating *PIK3CA* coverage in the overgrowth and PIK3CA panels. The regions covered by the PIK3CA and OVG panels are illustrated with the first and second tracts, respectively (PIK3CA in blue; OVG in red). Uniprot protein domains, ClinVar variants and COSMIC variants are also shown. (A) Full gene view (B) Zoomed in view excluding the 5’UTR, which was not covered by either panel.

A)


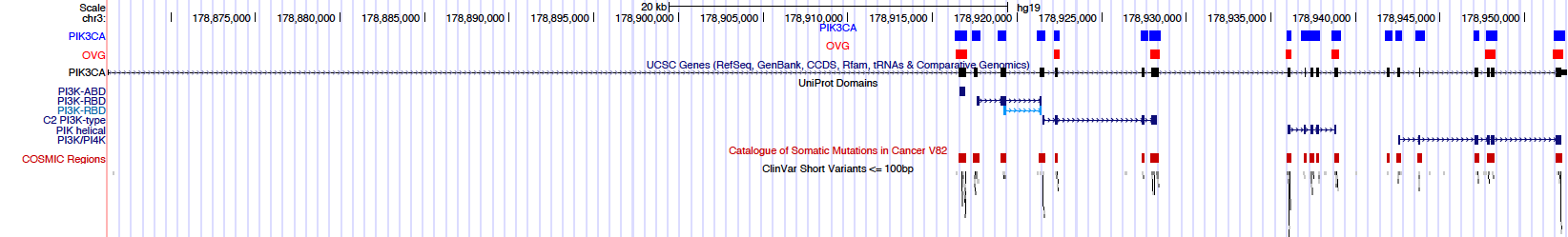


B)


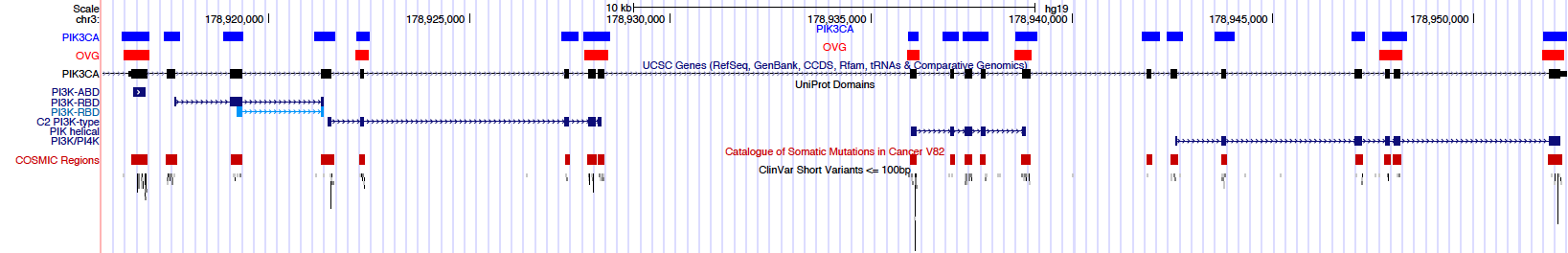

Supplement: Supplementary file 1 [file MGG3-7-na-s001.docx]
